# Association between ultra-processed food consumption and risk of irritable bowel syndrome and functional dyspepsia: a systematic review and meta-analysis of observational studies

**DOI:** 10.3389/fmed.2026.1780040

**Published:** 2026-03-24

**Authors:** Shengkai Li, Weikai Dong, Xi Chen, Xiaolong Tan

**Affiliations:** 1 Department of Emergency Surgery, Binzhou Medical University Hospital, Binzhou, Shandong Province, China; 2 Department of Cardiac Surgery, Cardiovascular Medical Center, Nanjing Drum Tower Hospital, Nanjing, Jiangsu Province, China; 3 Department of Gastrointestinal Surgery, Binzhou Medical University Hospital, Binzhou, Shandong Province, China

**Keywords:** diet, disorders of gut-brain interaction, functional dyspepsia, gastrointestinal health, irritable bowel syndrome, meta-analysis, NOVA classification, systematic review

## Abstract

**Background:**

Ultra-processed foods (UPFs) are increasingly prevalent in global diets and have been linked to various adverse health outcomes. However, evidence regarding their association with disorders of gut-brain interaction (DGBI), such as irritable bowel syndrome (IBS) and functional dyspepsia (FD), remains inconsistent.

**Objective:**

To systematically evaluate the association between UPFs consumption and the risk of IBS and FD through a comprehensive meta-analysis of observational studies.

**Methods:**

We conducted a systematic search of PubMed, Embase, Cochrane Library, and Web of Science from inception through December 2025. Observational studies assessing UPFs intake and clinically diagnosed IBS or FD in adults were included. Pooled effect estimates [odds ratios (OR)] were calculated using random-effects models. Heterogeneity was assessed with *I*^2^ statistics, and subgroup analyses were performed by exposure type and assessment method.

**Results:**

Ten studies (*N* = 232,448) were included. High UPFs consumption was significantly associated with higher odds of IBS (OR = 1.32, 95% CI: 1.16–1.49; based on 9 studies) and FD (OR = 1.26, 95% CI: 1.07–1.49; based on 3 studies). However, the FD finding was primarily driven by a single large cohort and lost statistical significance in sensitivity analysis, indicating the need for cautious interpretation. Subgroup analyses revealed the strongest association for carbonated/sugar-sweetened beverages (OR = 1.98, 95% CI: 1.40–2.80). Heterogeneity was substantially reduced in studies using standardized NOVA classifications or quartile-based comparisons (*I*^2^ = 0%). Sensitivity analyses supported robustness. The Egger test did not reveal significant funnel plot asymmetry for IBS outcomes (*p* = 0.153). Given the limited number of included studies (*n* = 9), this cannot definitively rule out the presence of publication bias.

**Conclusion:**

These findings suggest that reducing UPFs intake may be a promising dietary strategy for IBS prevention and management. For FD, the evidence is preliminary and requires confirmation in larger prospective studies.

**Systematic review registration:**

https://www.crd.york.ac.uk/prospero/display_record.php?ID=CRD420251269914, identifier PROSPERO (CRD420251269914).

## 1 Introduction

Irritable bowel syndrome (IBS) and functional dyspepsia (FD) are prevalent disorders of gut-brain interaction (DGBI) characterized by visceral hypersensitivity, motility disturbances, and gut-brain axis dysregulation, collectively imposing a substantial global healthcare burden (1). Though etiology is multifactorial, diet is recognized as a critical symptom trigger and an essential component of disease management (2). Global dietary shifts have driven a marked increase in ultra-processed foods (UPFs)—defined by the NOVA framework as extensively processed products (3). These UPFs are typically energy-dense, high in added sugars, low in fiber, and often contain additives such as emulsifiers and flavor enhancers. Given the rapid rise in UPFs consumption and their documented pro-inflammatory properties, quantifying their precise epidemiological role in IBS and FD risk is critical for developing targeted public health interventions (4).

UPFs disrupt intestinal homeostasis via multiple mechanisms. Specifically, industrial additives and artificial sweeteners compromise mucosal barrier integrity, thereby triggering microbial dysbiosis and low-grade inflammation, impairing gut-brain axis signaling (5). Furthermore, their high sugar load often exceeds the small intestine’s absorptive capacity, leading to increased colonic fermentation and consequent osmotic diarrhea (6). Systematic reviews confirm these alterations are linked to obesity, cardiovascular diseases, and elevated all-cause mortality (7). Conversely, epidemiological evidence regarding their contribution to disorders of gut-brain interaction remains notably heterogeneous (8, 9); while large cohort studies report significant associations, findings differ widely across specific subgroups and populations (10). This variation suggests that distinct food matrices may exert differential effects on gut health.

While the quantitative synthesis by Chen et al. (11) addresses broad health impacts of UPFs, evidence specifically concerning IBS and FD remains fragmented, confined to isolated discussions. Furthermore, existing individual studies are often constrained by cross-sectional designs or small sample sizes, thus impeding precise quantification of risk estimates. Notably, systematic analysis is lacking regarding how distinct UPFs consumption patterns differentially influence the risk of IBS and FD development. Therefore, this study conducts a systematic review and meta-analysis integrating recent observational evidence (including cross-sectional and prospective cohort studies) to precisely evaluate the UPFs intake-IBS/FD association, thereby providing robust evidence for clinical nutrition guidance and preventive strategies.

## 2 Materials and methods

### 2.1 Study registration and protocol

Conducted in accordance with the Preferred Reporting Items for Systematic Reviews and Meta-Analyses (PRISMA 2020) guidelines, the protocol for this systematic review and meta-analysis was registered prospectively with PROSPERO (CRD420251269914).

### 2.2 Search strategy

A comprehensive systematic search was executed across PubMed, Embase, the Cochrane Library, and Web of Science from inception through December 2025. The search strategy was designed using a combination of controlled vocabulary (MeSH terms) and free-text terms, categorized into two groups: (1) MeSH terms, including “Irritable Bowel Syndrome” and “Dyspepsia” (2) free-text terms, including “Ultra-processed” “NOVA classification” “highly processed,” “functional dyspepsia,” “functional gastrointestinal disorder*” and “fast food*.” Boolean operators (OR/AND) were used to integrate these concepts. To ensure high-quality evidence, we applied methodological filters to focus on observational study designs while excluding editorials and case reports. The complete, reproducible search strings for each individual database are provided in Supplementary Tables 1–4.

### 2.3 Eligibility criteria

Eligibility was strictly based on PICOS criteria: Studies required (1) a target population of general adults (≥18 years); (2) an exposure defined as UPFs (NOVA classification) (12) or the use of validated surrogate measures in studies lacking detailed dietary intake data. The exposure is widely employed in epidemiological research as a marker of highly processed food consumption, such as fast food, sugar-sweetened beverages, and canned foods; (3) a comparator of non-consumers or the lowest consumption category; and (4) outcomes restricted to definitive diagnoses of IBS or FD, confirmed by validated criteria (e.g., Rome criteria or physician diagnosis). Only observational designs (cross-sectional, case–control, cohort studies) were accepted. We excluded interventional trials (RCTs), reviews, case reports, editorials, conference abstracts, research on children or pregnant women, and studies reporting nonspecific gastrointestinal symptoms (e.g., bloating) without a confirmed diagnosis. Additionally, research focusing on broad dietary exposures lacking defined processing attributes (e.g., Western diet) was ineligible. To prevent duplication among overlapping cohorts, we prioritized the study with the largest sample size or the most detailed available data.

### 2.4 Study selection

Two reviewers (SL and WD) independently performed study selection according to predefined eligibility and exclusion criteria. The process began with title and abstract screening, where duplicate and irrelevant records were culled. Potentially eligible articles were subsequently retrieved for full-text assessment. Any disagreements were resolved through consultation with a third reviewer (XT), who served as the arbitrator.

### 2.5 Data extraction and quality assessment

Independent dual review screened titles, abstracts, and full texts (XC and WD). A standardized form facilitated data extraction, capturing: first author, publication year, country, study design, sample size, exposure definition, outcome diagnostic criteria, and effect estimates (Odds Ratios [OR], Relative Risks [RR], or Hazard Ratios [HR]) with 95% Confidence Intervals (CIs). Prioritizing adjusted models ensured the minimization of confounding bias. The adjusted model was predefined as one that adjusted for at least age, sex, and one additional metabolic factor. Studies only reporting crude or univariate associations were included but scrutinized during sensitivity analysis. Methodological quality for included observational studies was assessed using the Newcastle-Ottawa Scale (NOS). The NOS assigned stars based on three domains: selection of groups, comparability, and ascertainment of exposure/outcome. A score of ≥7 stars defined high quality. To maximize the utilization of available data, all identified studies were included in the initial meta-analysis, acknowledging variability in study quality. The potential influence of methodological rigor was subsequently and rigorously examined through leave-one-out sensitivity analyses to assess whether findings were disproportionately driven by studies of lower quality.

### 2.6 Statistical analysis

Statistical analyses utilized R software v. 4.5.1(13), specifically employing the “meta” package for the meta-analysis (14). We synthesized pooled risk estimates (OR, RR, and HR) to evaluate the association between UPFs consumption and IBS/FD risk. All effect estimates were log-transformed prior to pooling to normalize their distributions and stabilize variances. For studies reporting relative risk (RR) or hazard ratio (HR), where the incidence rate is less than or approximately 10%, the reported RR or HR will be treated as OR and incorporated into the meta-analysis for computation (15). Inter-study heterogeneity was assessed via Cochran’s Q test and the *I*^2^ statistic; significant heterogeneity was denoted by *p* < 0.10 or *I*^2^ > 50%. To account for expected clinical and methodological diversity and ensure a conservative estimate, a random-effects model was implemented. Between-study variance (tau^2^) was estimated using the Restricted Maximum Likelihood (REML) method, known for its robustness across varying sample sizes. Potential sources of heterogeneity were explored through subgroup analyses based on exposure category (e.g., Core UPFs, Fast Food/Junk Food, Beverages). Stability of pooled results was tested via sensitivity analyses, sequentially omitting individual studies. Publication bias was evaluated visually (funnel plots) and statistically (Egger’s regression test). Publication bias was evaluated visually (funnel plots) and statistically (Egger’s regression test). In accordance with Cochrane recommendations, these assessments were only performed for outcomes with at least 10 included studies, or where the number of studies was sufficient to provide a meaningful distribution. All inferences adopted a two-tailed significance threshold of *p* < 0.05.

## 3 Results

### 3.1 Literature retrieval results and basic characteristics

**Figure 1:**
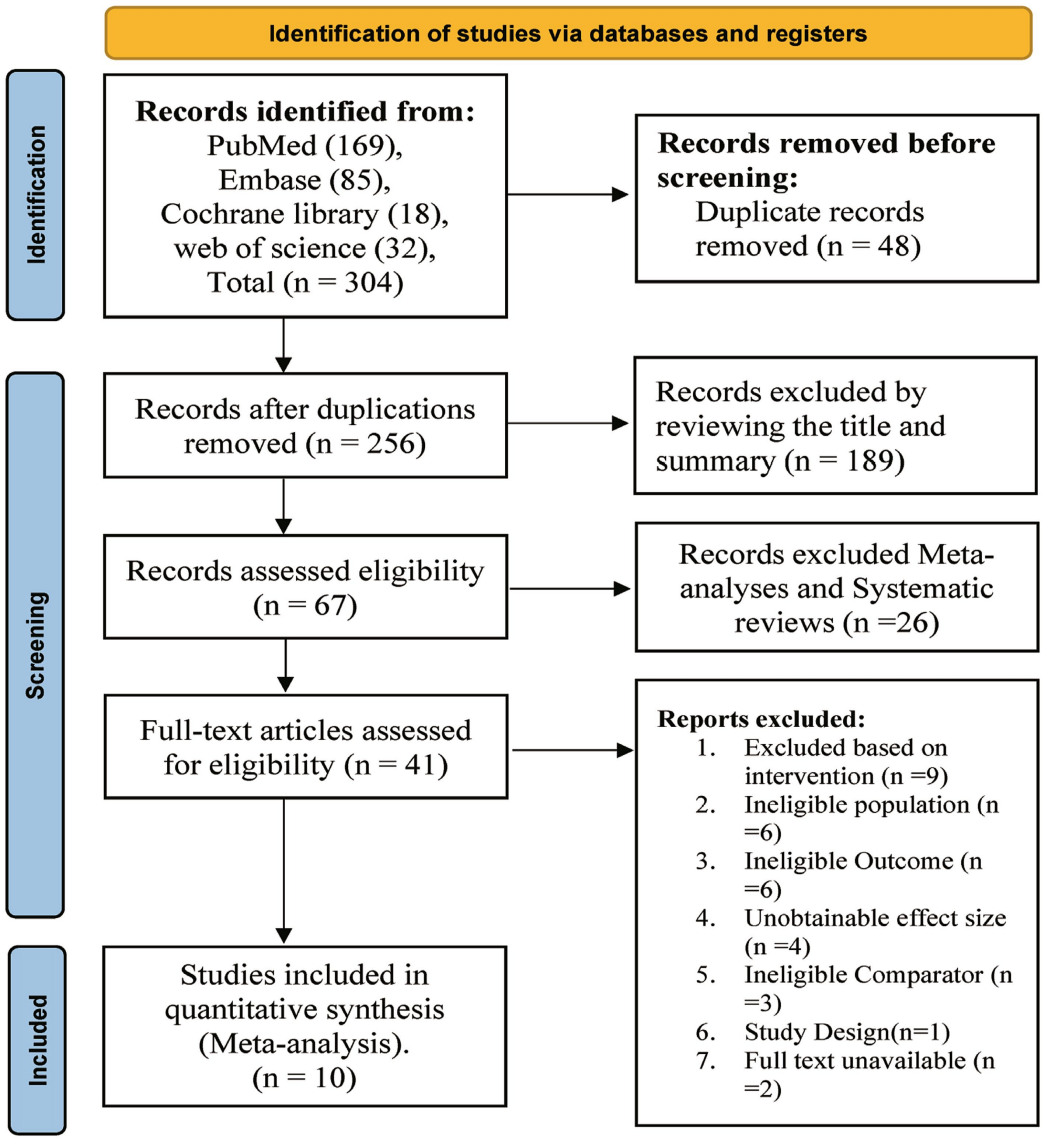
Literature search and selection process.

The systematic review process, detailed in Figure 1, began with a primary literature search, which yielded 304 records from four databases. After removing 48 duplicate records, initial screening excluded 189 publications based on title and abstract due to thematic irrelevance. An additional 26 records were then eliminated for being review articles. A detailed full-text assessment of the resulting 41 articles followed, leading to the inclusion of 10 studies for final analysis.

This systematic review synthesized 10 observational studies (total *N* = 232,448). Study designs comprised two large-scale prospective cohort studies (16, 17) and eight cross-sectional studies (8, 18–24). Sample sizes ranged from 184 to 178,711 participants. Geographically, the research spanned Europe (UK, France, Romania), the Middle East (Iran, Jordan, Yemen), and South Asia (India), supplemented by one multi-country dataset. Exposure assessment was heterogenous: three studies utilized the NOVA classification to define ultra-processed food (UPFs) consumption, while the remainder used specific proxy markers, specifically fast food (*n* = 4), carbonated/aerated beverages (*n* = 2), and canned food (*n* = 1). Outcomes focused primarily on irritable bowel syndrome (*n* = 9) and Functional Dyspepsia (FD, *n* = 3). The methodological quality of the included studies was generally moderate to high, with Newcastle-Ottawa Scale (NOS) scores ranging from 5 to 9 (Table 1).

**Table 1:** Main characteristics and data of each study.

Study (Author, Year)	Country	Design	Sample size (N)	Exposure definition	Outcome	Effect measure (95% CI)	Adjusted covariates	NOS score
Abdelshafi et al. (2025) (18)	Multi-country	Cross-sectional	8,275	Junk food (processed snacks, fast food)	IBS	OR 1.32 (0.99–1.75)	Age, Sex, BMI, Smoking, Activity, Stress	6
Jadallah et al. (2022) (8)	Jordan	Cross-sectional	1,094	Junk/Fast food	IBS	OR 0.81 (0.55–1.19)	Age, Sex, Smoking, Medications	7
Chirila et al. (2016) (19)	Romania	Cross-sectional	184	Canned Mixed Food	FD	OR 1.15 (0.39–3.43)	Univariate analysis only	8
Ghoshal et al. (2017) (20)	India	Cross-sectional	2,774	Aerated drinks	IBS	OR 1.87 (1.31–2.66)	Age, Sex, Dietary habits	8
Haghighatdoost et al. (2025) (21)	Iran	Cross-sectional	1892	Ultra-processed food (NOVA)	IBS/FD	OR 1.89 (1.10–3.55)/OR 1.73 (0.73–4.08)	Age, Sex, Energy, Activity, Smoking, SES	8
Khademolhosseini et al. (2011) (22)	Iran	Cross-sectional	1978	Fast food (Pizza, Sandwiches)	IBS	OR 1.52 (1.12–2.05)	Univariate analysis only	5
Khayyatzadeh et al. (2016) (24)	Iran	Cross-sectional	3,846	Fast food dietary pattern	IBS	OR 1.32 (0.99–1.75)	Age, Sex, BMI, Energy, Activity	8
Mahyoub et al. (2024) (24)	Yemen	Cross-sectional	351	Carbonated soft drinks	IBS	OR 3.35 (1.14–9.88)	Age, Sex, Khat chewing, Anxiety	7
Schnabel et al. (2018) (16)	France	Cohort	33,343	Ultra-processed food (NOVA)	IBS/FD	OR 1.25 (1.12–1.39)/HR 1.25 (1.05–1.47)	Age, Sex, BMI, Smoking, Alcohol, Diet quality	8
Wu et al. (2024) (17)	UK	Cohort	178,711	Ultra-processed food (NOVA)	IBS	HR 1.19 (1.07–1.33)	Age, Sex, BMI, Deprivation, Smoking, Diabetes	9

### 3.2 Primary outcomes of IBS risk

#### 3.2.1 Association between UPFs consumption and IBS risk

**Figure 2:**
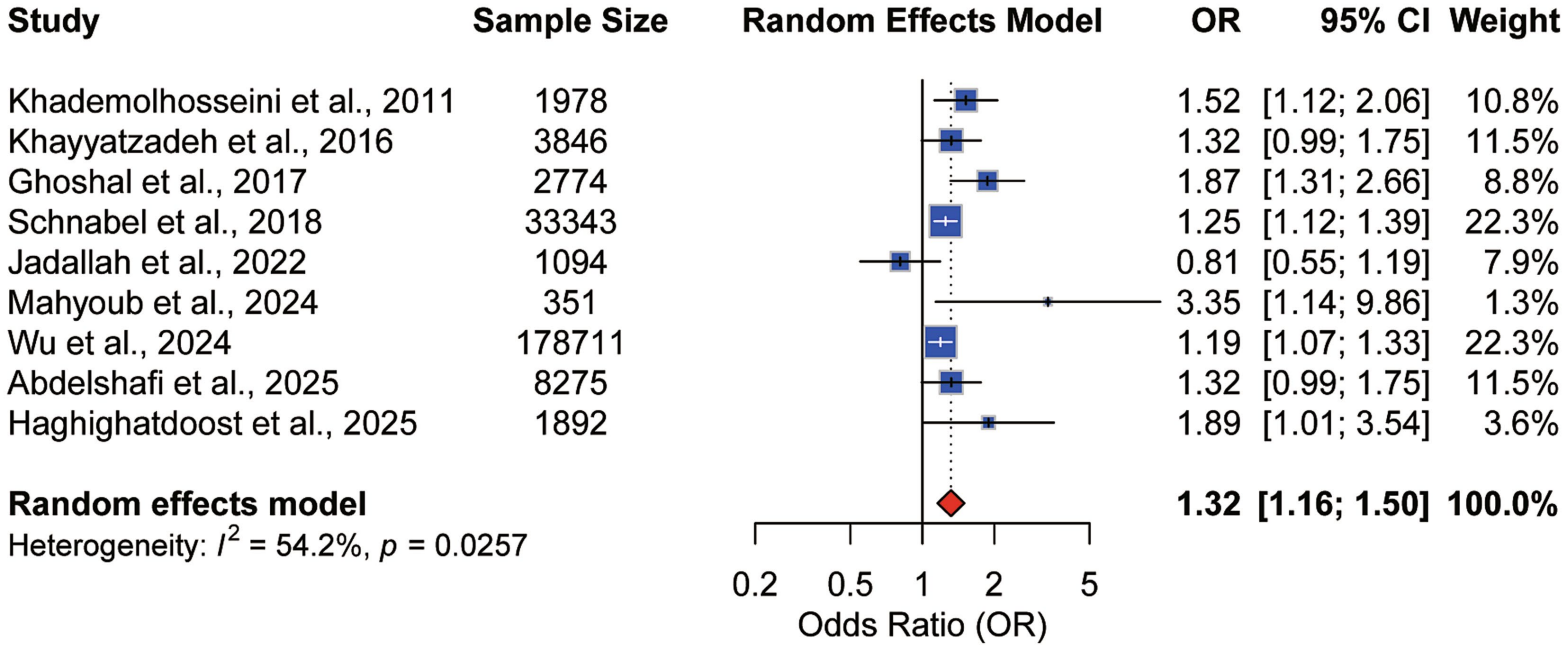
Association between UPFs consumption and IBS risk.

Nine observational studies (8, 16–18, 20–24) were integrated into the meta-analysis examining the association between ultra-processed food (UPFs) consumption and the risk of IBS. Employing a random-effects model, the pooled analysis revealed a significant positive association between high UPFs intake (including proxy markers such as fast food and sugary beverages) and increased IBS risk (Figure 2). The pooled effect size (ES) was 1.32 (95% CI: 1.16–1.49, *p* < 0.001), corresponding to a 32% greater risk of developing IBS for individuals in the highest consumption category relative to the lowest. This finding was accompanied by moderate heterogeneity (*I*^2^ = 54.2%, *p* = 0.026).

#### 3.2.2 Subgroup analysis of IBS risk

**Figure 3:**
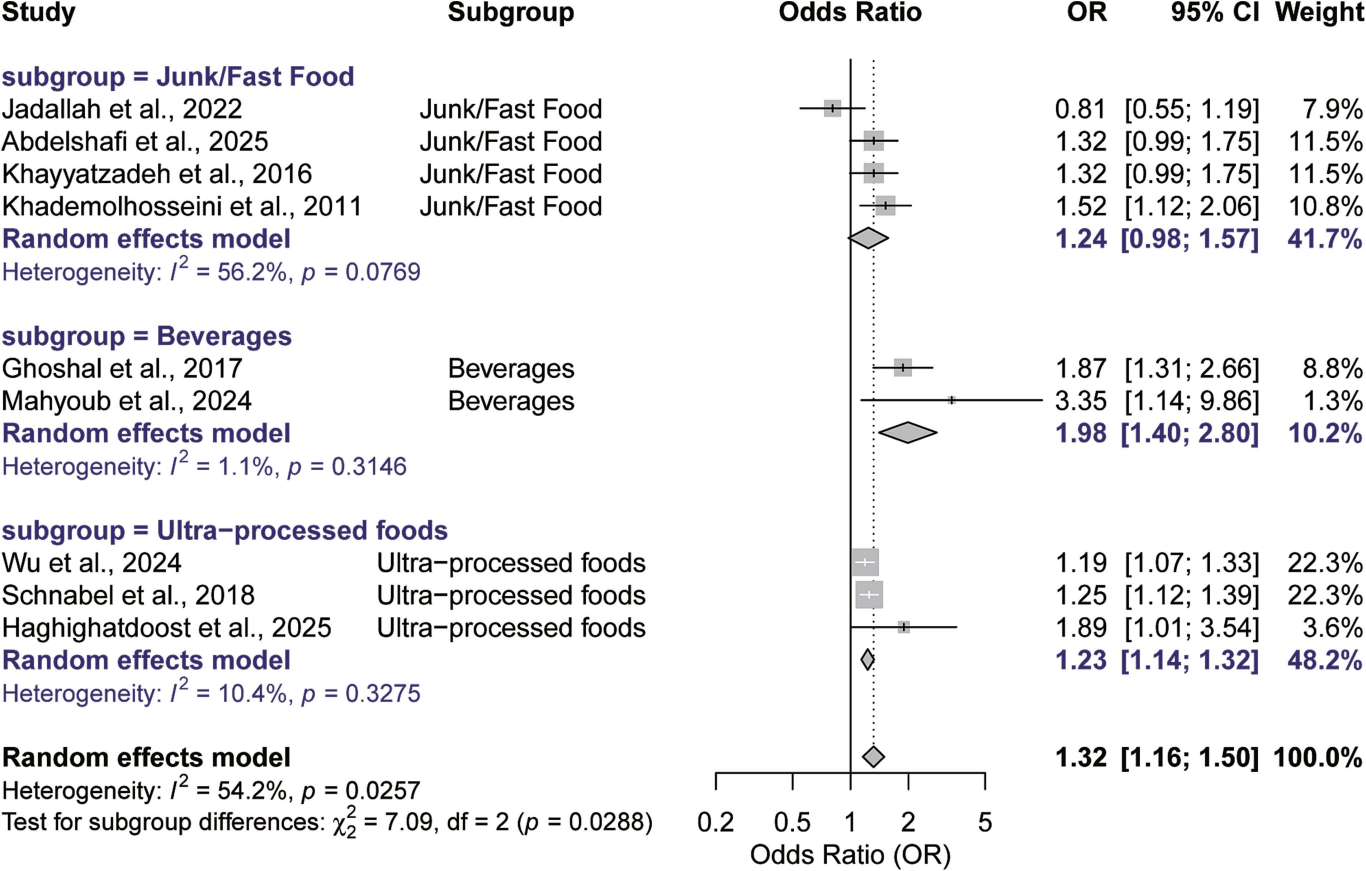
Subgroup analysis of the association between UPFs consumption and IBS risk by type of processed food. (Subgroups are defined based on the type of exposure investigated in the included studies: Junk/fast food, beverages, and ultra-processed foods).

Subgroup analyses were conducted to explore heterogeneity sources and assess finding robustness, stratifying results by exposure category and assessment method. Stratification by food type revealed distinct risk patterns. Notably, a separate analysis restricted to studies employing the standardized NOVA classification (*n* = 3) (16, 17, 21) yielded a robust and significant association (ES = 1.23, 95% CI: 1.13–1.34, *p* < 0.001) amidst low heterogeneity (*I*^2^ = 10.4%). This finding suggests that the association between UPFs and IBS may not be solely driven by specific foods, but persists even when applying the comprehensive NOVA framework. Conversely, investigations focused on carbonated or soft drinks (*n* = 2) (20, 24) yielded the strongest association: a pooled ES of 1.98 (95% CI: 1.40–2.80, *p* < 0.001) and minimal heterogeneity (*I*^2^ = 1.1%). In contrast, investigations of junk or fast food (*n* = 4) (8, 18, 22, 23) indicated a positive, yet non-significant, trend (ES = 1.24, *p* = 0.077), accompanied by moderate heterogeneity (*I*^2^ = 56.2%) (Figure 3).

**Figure 4:**
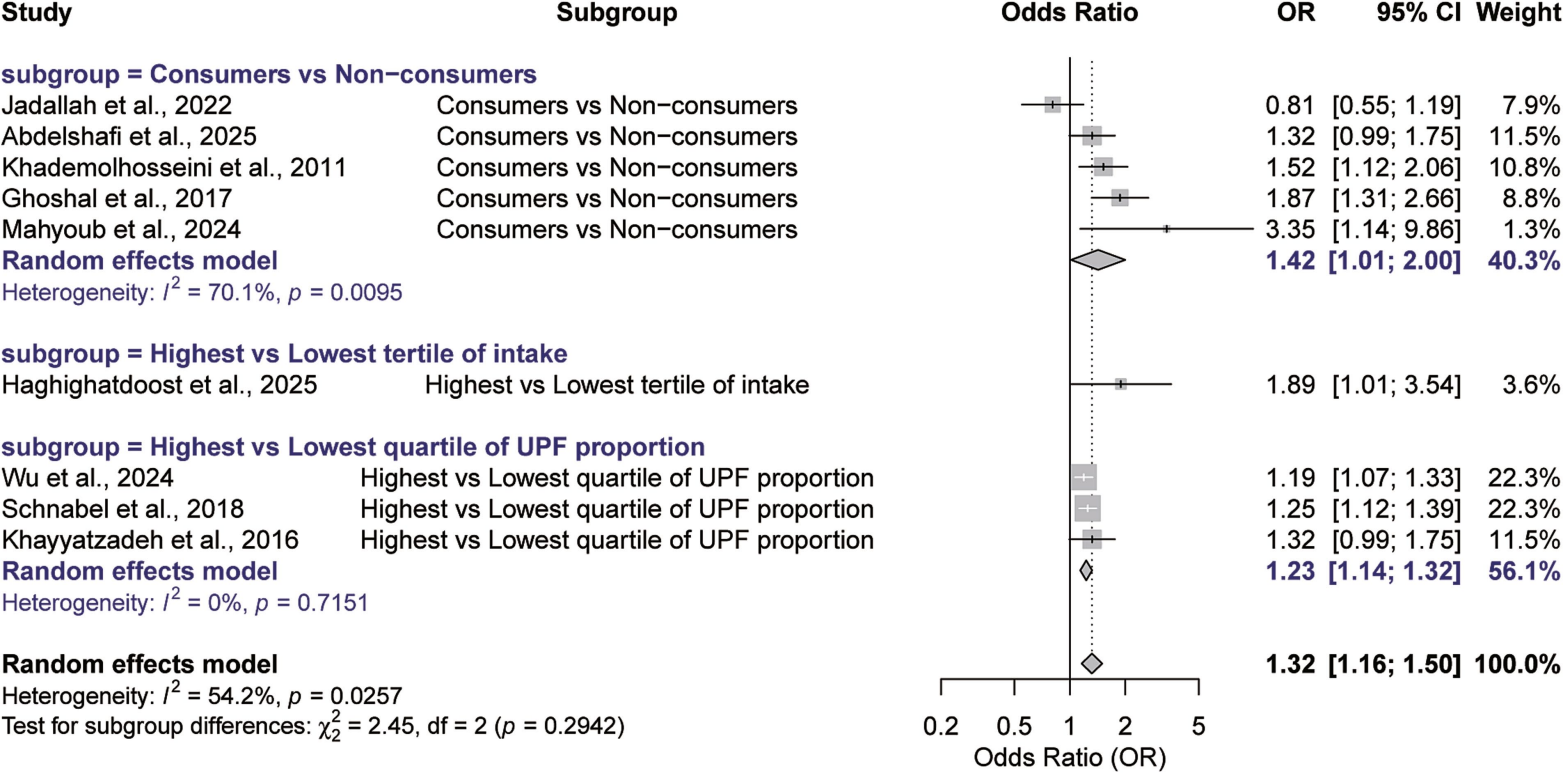
Subgroup analysis of the association between UPFs consumption and IBS risk according to the method of intake comparison. (Subgroups are defined based on how the exposure comparisons were reported in the original studies: consumers vs. non-consumers, highest vs. lowest tertile, and highest vs. lowest quartile).

Stratification by assessment method further emphasized the role of methodological rigor (Figure 4). Studies comparing the highest versus lowest quartiles of UPFs intake (*n* = 3) (16, 17, 23) yielded a highly significant and consistent result (ES = 1.23, 95% CI: 1.14–1.32, *p* < 0.001). No significant heterogeneity was detected in studies using standardized NOVA classifications or quartile-based comparisons (*I*^2^ = 0). This result suggesting that intake gradient analysis offers reliable evidence. In contrast, analyses based on a binary consumer/non-consumer comparison (*n* = 5) (8, 18, 20, 22, 24) reported a higher pooled estimate (ES = 1.42, 95% CI: 1.01–2.00, *p* = 0.032) but suffered from substantial heterogeneity (*I*^2^ = 70.1%).

#### 3.2.3 Sensitivity analysis and publication Bias

Sequential omission sensitivity analysis demonstrated the robustness of the pooled effect estimates (range: 1.24–1.37) (Supplementary Figure 1), confirming no single study disproportionately influenced the overall findings. Publication bias was evaluated visually via a funnel plot and quantitatively using Egger’s regression test. While the funnel plot exhibited relative symmetry around the pooled effect size (Supplementary Figure 2), its interpretation, along with that of the Egger regression test (*p* = 0.153), warrants considerable caution. This is primarily because the small number of included studies (*n* < 10) inherently limits both the plot’s ability to detect asymmetry and the test’s statistical power, thus precluding a definitive conclusion regarding the absence of publication bias.

### 3.3 Primary outcomes of FD risk

#### 3.3.1 Association between UPFs consumption and FD risk

The meta-analysis, which synthesized three observational studies assessing the link between UPFs consumption and FD risk, revealed a significant positive association (Supplementary Figure 3). The pooled effect estimate (ES), derived via a random-effects model, was 1.26 (95% CI: 1.07–1.49; *p* = 0.005), indicating a 26% increased FD risk in individuals categorized as high UPFs consumers. Critically, no heterogeneity was detected among the studies (*I*^2^ = 0, *p* = 0.757). However, these findings should be interpreted with caution due to the limited number of studies (*n* = 3).

#### 3.3.2 Sensitivity analysis and publication Bias

The limited sample size (*n* = 3) necessitated sensitivity analysis to assess result robustness. Excluding Chirila et al. (19) (pooled ES: 1.27; 95% CI: 1.07–1.49) or Haghighatdoost et al. (21) (pooled ES: 1.25; 95% CI: 1.06–1.47) yielded estimates virtually unchanged from the overall figure, confirming stability. Conversely, removal of the large-scale Schnabel et al. (16) cohort (*n* = 33,343) caused a loss of statistical significance (ES = 1.48; 95% CI: 0.75–2.91), demonstrating that this high-quality, large-sample study primarily drives the overall significant finding (Supplementary Figure 4). The primary significant finding appears predominantly driven by a single large study, rendering the evidence for an association between UPFs and FD preliminary. Publication bias for FD was not assessed using funnel plots or Egger’s test due to the insufficient number of included studies (*n* = 3), as these tests lack the statistical power to provide reliable inferences with fewer than 10 studies (25).

## 4 Discussion

This systematic review and meta-analysis is the inaugural application of the NOVA framework to evaluate the link between ultra-processed food (UPFs) intake and the onset of IBS and FD. Synthesizing 10 observational studies (*N* > 230,000) across Europe, the Middle East, and Asia, our analysis robustly demonstrates that high UPFs consumption is associated with disorders of gut-brain interaction, independent of several potential confounders, increasing the risk of IBS by 32% (OR = 1.32, 95% CI: 1.16–1.49) and FD by 26% (OR = 1.26, 95% CI: 1.07–1.49). It is important to note that when analyses were restricted to studies employing strict quartile comparisons, heterogeneity disappeared (*I*^2^ = 0). While this observation indicates that inconsistencies in the extant literature are predominantly attributable to methodological discrepancies in exposure classification, it does not preclude the potential influence of other variables. The clinical diversity observed across different regions (for example, between the Middle East and Europe) and the variations in IBS and FD diagnostic criteria may interact with dietary assessment methods, thereby influencing the observed risk estimates.

Prior to NOVA’s widespread adoption, DGBI epidemiological research focused on macronutrients (e.g., fats, carbohydrates) (26, 27) or broader dietary patterns (28). Although these studies linked low-quality diets to exacerbated IBS symptoms, findings based on proxy indicators such as fast food or fried foods often remained inconsistent and inconclusive (29, 30). Our own data mirrored this ambiguity; for instance, the fast food subgroup exhibited a non-significant trend (*p* = 0.074), likely attributable to heterogeneous cultural definitions. Our meta-analysis reconciles these discrepancies by highlighting the pivotal role of exposure definition and assessment methodologies. Subgroup analyses revealed significant heterogeneity (*I*^2^ = 70.1%) and marginal statistical significance in studies employing vague definitions (e.g., junk food) or simplistic binary comparisons (consumers vs. non-consumers). studies leveraging the standardized NOVA classification, focusing on core UPFs, yielded highly consistent and significant results (*I*^2^ = 10.4%, *p* < 0.001). Although some studies employed proxy exposures like fast food or beverages, these are not strictly equivalent to NOVA-defined UPFs. Such proxies can omit true UPFs (e.g., packaged breads, breakfast cereals) while inadvertently including minimally processed items (e.g., homemade fast food). Nonetheless, the consistency between our NOVA-restricted analysis and broader pooled estimates indicates a reliable underlying association. Heterogeneity was virtually eliminated (*I*^2^ = 0%) when exposure assessment employed a rigorous quantitative gradient (e.g., intake quartiles). These results suggest that previous literature inconsistencies may arose from variations in categorization and assessment methods. This outcome aligns with a recent umbrella review linking UPFs consumption to diverse adverse health outcomes (including mental and metabolic disorders) (31), underscoring the necessity of shifting the DGBI etiology framework from a nutrient-centric to a food-processing-centric perspective.

Ultra-processed foods (UPFs) intake is linked to an increased risk of disorders of gut-brain interaction (DGBI) via multiple synergistic mechanisms. Food additives, often acting as a chemical mixture, disrupt the intestinal barrier and provoke inflammation (32). For example, artificial color Sunset Yellow inhibits intestinal epithelial repair and elicits oxidative stress in animal models (33), its long-term intake disrupts gut microbiota composition and reduces jejunal E-cadherin/*β*-catenin and Trefoil factor (TFF)-3 expression, thereby impairing intestinal barrier function (34). Additionally, thickeners like polysorbate-80 (P80) compromise the intestinal mucosal barrier and diminish beneficial gut bacteria abundance (35). UPFs, characterized as acellular nutrients due to their lack of a natural cellular matrix, promote rapid proximal intestinal nutrient absorption (36). This leads to insufficient fermentable dietary fiber reaching the distal colon (37), and concurrent nutrient enrichment in the proximal intestine. This distinct absorption pattern can induce gut microbiota dysbiosis (38), reducing beneficial bacterial metabolite synthesis, including short-chain fatty acids (SCFAs) (39). The reduction in SCFAs is critical, as they nourish colonic epithelial cells, modulate the enteric nervous system, and maintain intestinal barrier tight junction integrity (40).

High UPFs intake, however, often co-occurs with several confounding lifestyle factors: reduced dietary fiber intake, elevated smoking rates, diminished physical activity, lower socioeconomic status, and increased psychological distress (7). This clustering poses a methodological challenge, complicating the disentanglement of UPFs-specific effects from broader unhealthy lifestyle impacts. While most studies in this review adjusted for basic demographics and smoking, few controlled for psychological comorbidities or overall dietary quality. Psychological distress, independently associated with unhealthy dietary choices and DGBI pathogenesis (41), further suggests these confounders could yield a spurious UPF-DGBI association. Moreover, the substitution hypothesis (42) posits that UPFs risk stems not from processing perse, but from their high caloric density and low nutritional diversity displacing protective nutrients (e.g., dietary fiber, polyphenols). Cross-sectional evidence also merits consideration for reverse causality, where symptomatic individuals might perceive UPFs as triggers, potentially leading to reduced consumption. Of the 10 studies reviewed, eight were cross-sectional (concurrently assessing exposure and outcomes), with only two prospective cohort studies identified. Notably, both prospective studies reported effect estimates (HR = 1.19–1.25) consistent with cross-sectional findings, suggesting reverse causality alone is an improbable sole explanation for the observed association.

The subgroup analysis identified that carbonated/sugar-sweetened beverages carried the highest risk estimate (OR = 1.98). This pronounced effect likely stems from excessive fructose and high-fructose corn syrup (HFCS) intake. When fructose intake exceeds small intestinal absorptive capacity, it undergoes colonic fermentation, generating gas and elevating osmotic pressure (43). This mechanism directly triggers symptoms of bloating and abdominal pain. Non-caloric artificial sweeteners (NAS), including aspartame, acesulfame potassium (Ace-K), advantame, saccharin, and sucralose, are commonly used in foods and beverages. In Caco-2 cells, saccharin was found to compromise intestinal barrier integrity by promoting the ubiquitination of claudin-1 via activation of NF-κB (44). Guo et al. (45) demonstrated that sucralose triggers the TLR5/MyD88/NF-κB signaling pathway, leading to increased production of IL-1β, IL-17A, IL-18, and TNF-α. Such systemic inflammation may traverse the circulation to affect the central nervous system, thereby exacerbating dysregulation of the gut-brain axis. This mechanism may also explain the high prevalence of anxiety and depression observed among high UPFs consumers, aggravating DGBI symptoms.

Despite the limited sample (*N* = 3) of studies investigating functional dyspepsia (FD), the pooled analysis demonstrated a statistically significant association (OR = 1.26) accompanied by negligible heterogeneity (*I*^2^ = 0%). However, sensitivity analysis revealed this finding was primarily driven by the large-scale NutriNet-Santé cohort (16). This dependence on a single dominant study thus mandates large-scale prospective replication research. The primary reliance of our FD analysis on a single large cohort mandates cautious interpretation of its findings. Current evidence for functional dyspepsia remains preliminary and hypothesis-generating, rather than definitive. Therefore, these findings necessitate validation through large-scale prospective cohort studies employing standardized UPFs assessment. Despite these limitations, the biological plausibility of an association between UPFs and FD is supported by mechanistic evidence. FD shares pathophysiological pathways with IBS, including visceral hypersensitivity, low-grade inflammation, and gut-brain axis dysregulation (46). These mechanisms suggests that the detrimental effects of food processing may extend to the gastroduodenal region.

These findings may have implications for clinical practice and public health policy, though confirmation in prospective trials is needed. Clinically, although the low-FODMAP diet is evidence-supported for managing IBS (47), its practical complexity often limits patient adherence. Promoting the simpler directive: reduce UPFs intake, offers a potentially practical and accessible adjunctive dietary strategy for managing DGBI. Publicly, the dose–response relationship between UPFs consumption and DGBI risk (16), confirmed by this analysis and existing cohort studies, provides a robust rationale for intervention. This evidence supports implementing public health policies focused on reducing UPFs consumption, which represents a promising strategy for lowering the incidence of IBS and functional dyspepsia.

## 5 Limitations

This study is subject to several limitations. The primary constraint pertains to study design: although two large prospective cohorts support temporality (16, 17), the majority of included studies were cross-sectional, precluding definitive causal inferences and rendering the results susceptible to reverse causation bias. Outcome assessment exhibited heterogeneity. Diagnosis of IBS and FD relied on disparate Rome criteria versions (II, III, or IV). A primary limitation concerned the pooling of different effect measures; specifically, treating OR, RR, and HR as equivalent may have overestimated true relative risk in high-prevalence settings. Future research should thus prioritize RR or HR inclusion for more precise risk quantification. Moreover, publication bias assessment was limited by the sparse number of eligible studies. Hence, non-significant Egger’s test results and visual funnel plot symmetry do not definitively establish bias absence but rather suggest no overt bias was detectable within the current analytical framework. Residual confounding remains a salient concern. Despite controlling for common covariates (e.g., age, sex, BMI, smoking), critical factors like psychological factors and socioeconomic factors were inconsistently accounted for, potentially introducing bias into the observed associations.

## 6 Conclusion

Our systematic review and meta-analysis establish a strong, independent association between high UPFs consumption and elevated IBS risk. For FD, a potential positive association is suggested; however, the evidence remains preliminary due to a limited number of studies and reliance on a single major cohort. Therefore, while UPFs reduction offers a promising strategy for IBS management, its role in FD prevention warrants further investigation through large-scale prospective trials. The association was particularly strong regarding carbonated/sugar-sweetened beverages, and its consistency persisted when applying standardized NOVA definitions and rigorous quantitative assessments. Therefore, UPFs reduction may represent a promising dietary strategy for the prevention and management of DGBI, pending confirmation from interventional studies.

## Data Availability

The original contributions presented in the study are included in the article/Supplementary material, further inquiries can be directed to the corresponding author.
