# Supplementary material for: Association between ultra-processed food consumption and risk of irritable bowel syndrome and functional dyspepsia: a systematic review and meta-analysis of observational studies
Source: Front Med (Lausanne). 2026 Mar 24;13:1780040. doi: 10.3389/fmed.2026.1780040 (PMC13053308; doi:10.3389/fmed.2026.1780040)
Supplement: Supplementary file 1 [file Table_1.DOCX]

Supplementary Material

# Supplementary Figures and Tables

For more information on Supplementary Material and for details on the different file types accepted, please see [here](https://www.frontiersin.org/guidelines/author-guidelines#supplementary-material).

## Supplementary Figures


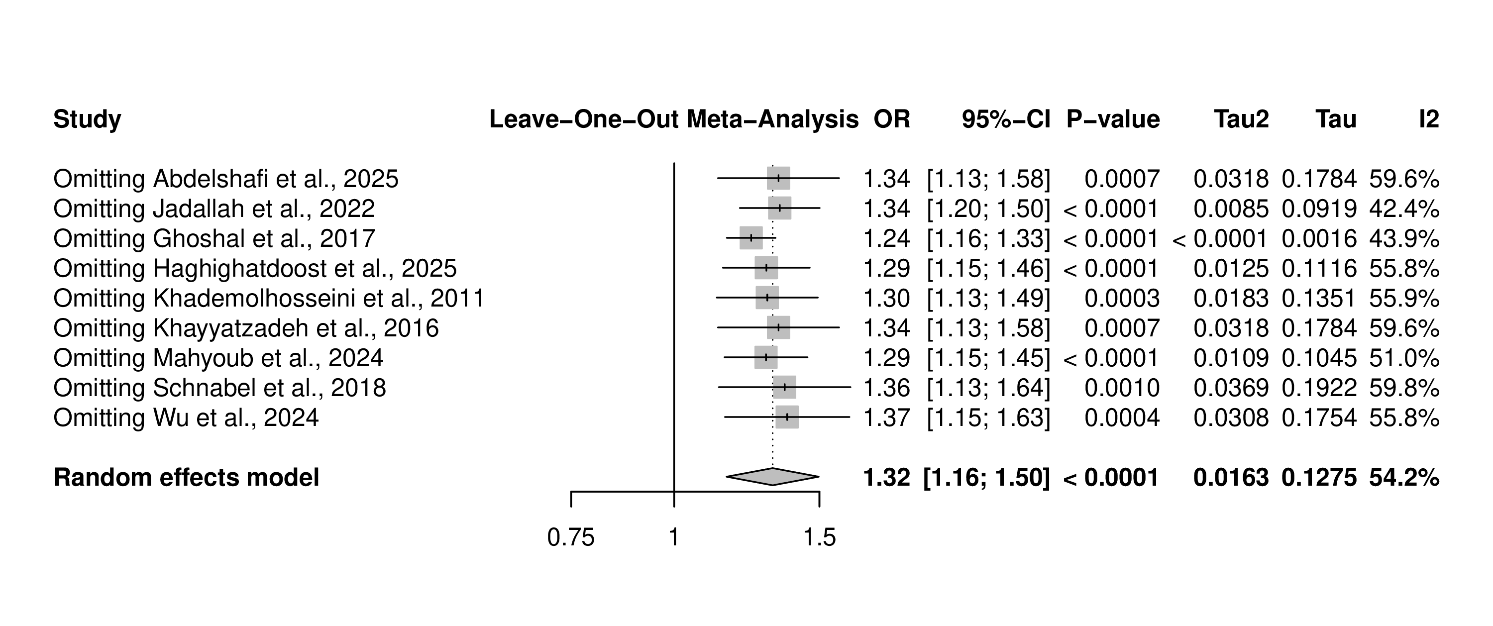


**Supplementary Figure 1.** Leave-one-out sensitivity analysis of the association between UPFs consumption and IBS risk.





**Supplementary Figure 2.** Funnel plot for the evaluation of publication bias in studies investigating the association between UPFs consumption and IBS risk.


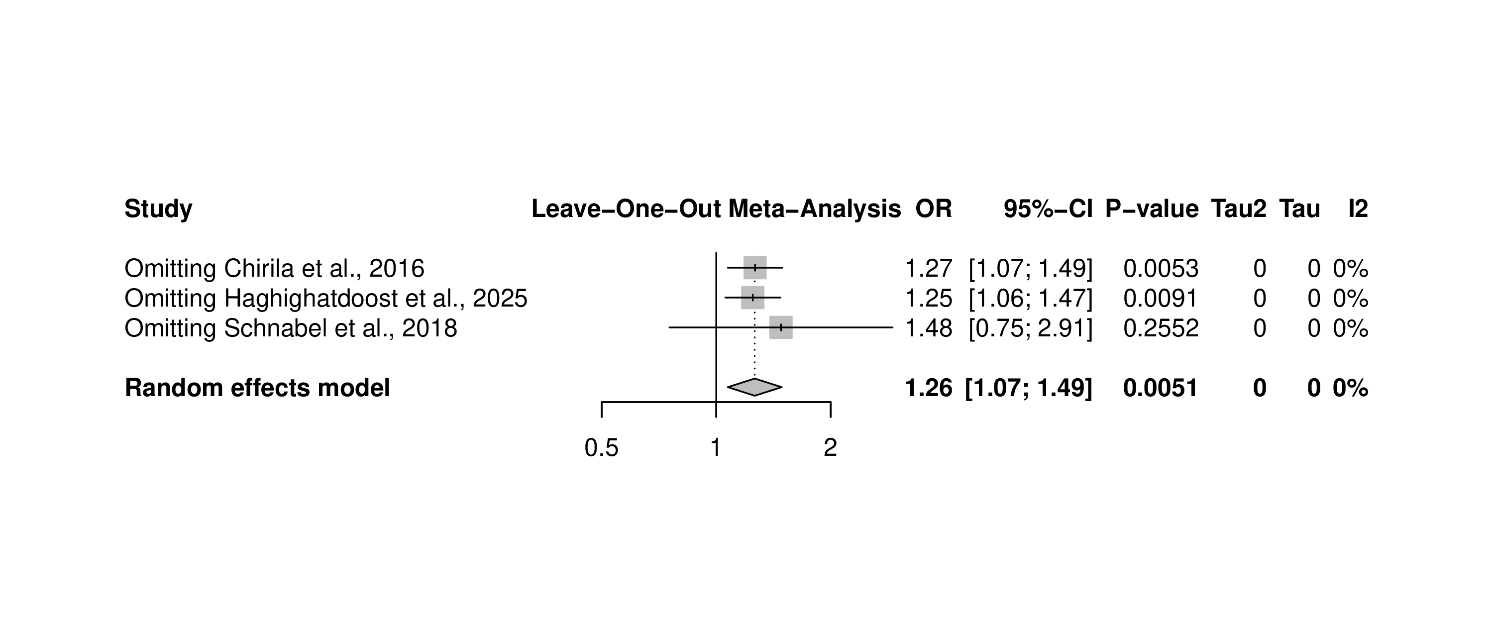


**Supplementary Figure 3.** Leave-one-out sensitivity analysis of the association between UPFs consumption and FD risk.

**Supplementary Table 1.** Detailed search strategies for PubMed.

| Pubmed |  |  |
| --- | --- | --- |
| NO. | CONCENT | RESULT |
| #1 | Search: ("Ultra-processed"[Title/Abstract] OR "ultraprocessed"[Title/Abstract] OR "NOVA classification"[Title/Abstract] OR "highly processed"[Title/Abstract] OR "industrial* food*"[Title/Abstract] OR "convenience food*"[Title/Abstract] OR "fast food*"[Title/Abstract] OR "junk food*"[Title/Abstract] OR "soft drink*"[Title/Abstract] OR "sugar-sweetened beverage*"[Title/Abstract] OR "western diet"[Title/Abstract] OR "western dietary pattern"[Title/Abstract]) | 24948 |
| #2 | Search: ("Irritable Bowel Syndrome"[MeSH Terms] OR "irritable bowel"[Title/Abstract] OR "IBS"[Title/Abstract] OR "Dyspepsia"[MeSH Terms] OR "functional dyspepsia"[Title/Abstract] OR "indigestion"[Title/Abstract] OR "Gastrointestinal Diseases/psychology"[MeSH Terms] OR "functional gastrointestinal disorder*"[Title/Abstract] OR "FGID"[Title/Abstract] OR "DGBI"[Title/Abstract] OR "Rome III"[Title/Abstract] OR "Rome IV"[Title/Abstract] OR "abdominal pain"[Title/Abstract] OR "bloating"[Title/Abstract] OR "constipation"[Title/Abstract] OR "diarrhea"[Title/Abstract]) | 240609 |
| #3 | #1 AND #2 | 169 |

**Supplementary Table 2.** Detailed search strategies for Embase.

| Embase |  |  |
| --- | --- | --- |
| NO. | CONCENT | RESULT |
| #1 | 'ultraprocessed food'/exp OR 'ultraprocessed food' OR 'ultraprocessed food':ti,ab OR 'ultra processed food':ti,ab OR 'nova classification':ti,ab OR 'highly processed':ti,ab OR 'industrial* food*':ti,ab OR 'convenience food*':ti,ab OR 'junk food':ti,ab OR 'fast food':ti,ab OR 'soft drink':ti,ab OR 'sugar sweetened beverage':ti,ab | 17467 |
| #2 | ('irritable colon'/exp OR 'irritable bowel':ti,ab OR 'IBS':ti,ab OR 'dyspepsia'/exp OR 'functional dyspepsia':ti,ab OR 'indigestion':ti,ab OR 'functional gastrointestinal disorder':ti,ab OR 'FGID':ti,ab OR 'disorders of gut brain interaction':ti,ab OR 'DGBI':ti,ab OR 'Rome III':ti,ab OR 'Rome IV':ti,ab) | 94028 |
| #3 | #1 AND #2 | 85 |

**Supplementary Table 3.** Detailed search strategies for Web of Science.

| Web of Science |  |  |
| --- | --- | --- |
| NO. | CONCENT | RESULT |
| #1 | TS=("Ultra-processed" OR "ultraprocessed" OR "NOVA classification" OR "highly processed" OR "industrial* food*" OR "fast food*" OR "junk food*" OR "soft drink*" OR "sugar-sweetened beverage*") | 29519 |
| #2 | TS=("Irritable Bowel Syndrome" OR "irritable bowel" OR "IBS" OR "Dyspepsia" OR "functional dyspepsia" OR "functional gastrointestinal disorder*" OR "FGID" OR "DGBI" OR "Rome III" OR "Rome IV") | 53450 |
| #3 | #1 AND #2 | 74 |

**Supplementary Table 4.** Detailed search strategies for Cochrane Library.

| Cochrane |  |  |
| --- | --- | --- |
| NO. | CONCENT | RESULT |
| #1 | [mh "Irritable Bowel Syndrome"] OR [mh "Dyspepsia"] | 3300 |
| #2 | ("irritable bowel" OR "IBS" OR "functional dyspepsia" OR "indigestion" OR (functional NEXT gastrointestinal NEXT disorder*) OR "FGID" OR "disorders of gut brain interaction" OR "DGBI" OR "Rome III" OR "Rome IV"):ti,ab,kw | 9321 |
| #3 | #1 OR #2 | 10072 |
| #4 | ("ultra-processed" OR "ultraprocessed" OR "NOVA classification" OR "highly processed" OR (industrial* NEXT food*) OR (fast NEXT food*) OR (junk NEXT food*) OR (soft NEXT drink*) OR (sugar NEXT sweetened NEXT beverage*)):ti,ab,kw | 2743 |
| #5 | #3 AND #4 | 18 |
